# Losing a child: finding meaning in bereavement

**DOI:** 10.3402/ejpt.v5.22910

**Published:** 2014-03-31

**Authors:** Julia Bogensperger, Brigitte Lueger-Schuster

**Affiliations:** Faculty of Psychology, University of Vienna, Vienna, Austria

**Keywords:** Complicated grief, posttraumatic growth, death, parental bereavement, meaning reconstruction

## Abstract

**Background:**

Confronting the loss of a loved one leads us to the core questions of human existence. Bereaved parents have to deal with the rupture of a widely shared concept of what is perceived to be the natural course of life and are forced into meaning reconstruction.

**Objective:**

This study aims to expand upon existing work concerning specific themes of meaning reconstruction in a sample of bereaved parents. More specifically, the relationship between meaning reconstruction, complicated grief, and posttraumatic growth was analyzed, with special attention focused on traumatic and unexpected losses.

**Method:**

In a mixed methods approach, themes of meaning reconstruction (sense-making and benefit-finding) were assessed in in-depth interviews with a total of 30 bereaved parents. Posttraumatic growth and complicated grief were assessed using standardized questionnaires, and qualitative and quantitative results were then merged using data transformation methods.

**Results:**

In total 42 themes of meaning reconstruction were abstracted from oral material. It was shown that sense-making themes ranged from causal explanations to complex philosophical beliefs about life and death. Benefit-finding themes contained thoughts about personal improvement as well as descriptions about social actions. Significant correlations were found between the extent of sense-making and posttraumatic growth scores (*r*_s_=0.54, *r*_s_=0.49; *p*<0.01), especially when the death was traumatic or unexpected (*r*_s_=0.67, *r*_s_=0.63; *p*<0.01). However, analysis revealed no significant correlation with complicated grief. Overall results corroborate meaning reconstruction themes and the importance of meaning reconstruction for posttraumatic growth.

The loss of a child is one of the most devastating experiences for a parent and is associated with diverse maladaptive developments (Znoj, 2004). Bereaved parents are at risk of anxiety disorders, depression (Kreicbergs, Valdimarsdóttir, Onelöv, Henter, & Steineck, 2004; Rogers, Floyd, Seltzer, Greenberg, & Hong, 2008), complicated grief (Kersting, Brähler, Glaesmer, & Wagner, 2011), psychiatric hospitalization (Li, Laursen, Precht, Olsen, & Mortensen, 2005), and mortality (Li, Precht, Mortensen, & Olsen, 2003). Despite these alarming results, research dealing with bereaved parents is still rare (Rogers et al., 2008).

Losing a child is recognized as especially disruptive and challenging to the expected order of life events: The young ought to outlive the old. This inconsistency of the loss with the pre-loss worldview brings upon fundamental questions of meaning in life (Davis, Nolen-Hoeksema, & Larson, 1998; Keesee, Currier, & Neimeyer, 2008; Lichtenthal, Currier, Neimeyer, & Keesee, 2010; Murphy, Johnson, & Lohan, 2003; Polatinsky & Esprey, 2000; Wheeler, 2001). Hence, a process of finding and integrating meaning of the loss and its consequences into one's worldview takes place. Neimeyer (2000) defines this “meaning reconstruction,” as sense-making, benefit-finding, and identity-change (Gillies & Neimeyer, 2006).

Sense-making refers to those thinking processes engaged in to understand the loss by incorporating it into a personal worldview, for example, by understanding the cause of death. Benefit-finding refers to the process of discovering positive consequences in the face of adversity, like increased compassion (Davis et al., 1998). In the current study, identity-change is conceptualized as an outcome of meaning reconstruction rather than a process itself. This goes along with the majority of authors who conceptualize the positive changes in one's identity in the aftermath of adversity as posttraumatic growth (Tedeschi & Calhoun, 1996).

Similar to the concept of meaning reconstruction, the “Model of Growth in the Context of Grief” (Calhoun, Tedeschi, Cann, & Hanks, 2010) states that inconsistency of the loss with pre-loss worldviews triggers intense cognitive work to reconstruct a new worldview incorporating the loss in a meaningful way (Calhoun et al., 2010; Cann et al., 2010). Thus, meaning reconstruction describes a process and posttraumatic growth describes an outcome, both concepts concern the integration of the loss (Calhoun et al., 2010; Gillies & Neimeyer, 2006). Therefore, a positive relationship between meaning reconstruction and posttraumatic growth would be expected; however studies show heterogeneous results: Some studies showed that benefit-finding is correlated with posttraumatic growth, whereas thoughts about the meaning of life (part of the sense-making process) are not necessarily (Cadell, Regehr, & Hemsworth, 2003; Engelkemeyer & Marwit, 2008; Hogan & Schmidt, 2002). More recent research suggests that while the presence of meaning in life is associated with posttraumatic growth (Linley & Joseph, 2011; Triplett, Tedeschi, Cann, Calhoun, & Reeve, 2012), the search for meaning is associated with negative change (Linley & Joseph, 2011). However, to date research analyzing the relations of the two concepts is scarce, and often only parts of meaning reconstruction are analyzed.

As stated above, bereaved parents have a high risk of complicated grief, especially those bereaved by traumatic causes (Keesee et al., 2008; Wijngaards-de Meij et al., 2005). Complicated grief is characterized by symptoms of intense grief persisting for more than 6 months (Prigerson et al., 1995). In contrast, individuals with non-complicated grief gradually adapt to the reality of the loss and over time the intensity of grief declines (Znoj, 2004). Adaptation is possible through integration of the loss and its consequences into the autobiographic memory and the worldview. That is meaning reconstruction. But then the intense emotional distress of individuals with complicated grief hinders the cognitive work required for meaning reconstruction (Boelen, van den Hout, & van den Bout, 2006). Recent empirical work supports the notion that the inability to find any meaning in a loss is associated with distress and complicated grief (Bonanno et al., 2007; Davis et al., 1998; Keesee et al., 2008; Lichtenthal et al., 2010), whereas positive meaning reconstruction is negatively correlated with complicated grief (Currier, Holland, & Neimeyer, 2006).

With a mixed methods study, Lichtenthal et al. (2010) identified specific themes of meaning reconstruction in a sample of bereaved parents with qualitative methods. The present study aims to scrutinize and expand these. Further, associations of meaning reconstruction with complicated grief and posttraumatic growth are analyzed, drawing special attention to violent and unexpected losses.

## Method

### Procedures and participants

Bereaved parents were invited to participate in our study via bereavement support groups and psychotherapists. Study aims and procedures were described, and contact details were submitted in the form of a letter. Study inclusion required that the time since the loss occurred exceeded a year, given that meaning reconstruction (Murphy et al., 2003) as well as posttraumatic growth (Taku, Calhoun, Cann, & Tedeschi, 2008; Taku, Cann, Tedeschi, & Calhoun, 2009) and the development of complicated grief (Prigerson et al., 2009) take time. Furthermore, to account for the fact that perinatal bereavement poses a different challenge in meaning reconstruction (Uren & Wastell, 2002), the deceased child had to have been born rather than died in the womb. A total sample comprised *N*=31 bereaved parents. Two participants decided to answer the interview questions in writing instead of orally. Among these two, one participant had to be excluded due to missing data. Another participant was box-plot identified as an outlier and therefore remained excluded from quantitative analysis exclusively.

The mean age was 51.0 years (*SD*=11.5 years; range=29–72 years). There were 21 (70%) women and nine (30%) men. Of the 30 participants, most were married (66.7%), some divorced (13.3%), 6.7% were single, 13.3% in a relationship and none were widowed. More than half of the participants (*N=*18) stemmed from the same parental dyad. Fifty percent of the parents reported that their child died of an illness, 30% had lost their child through an accident, 16.7% were bereaved due to suicide and 3.3% by homicide. Consequently, 50% of the deaths were coded as traumatic, and 50% as non-traumatic (objective measure). Nonetheless, only 16.7% of the parents reported having subjectively anticipated the loss; most (83.3%) did not (subjective measure). The mean length of time since the loss had occurred was 9.73 years (*SD*=7.8; range=1–26). Participants from the same (*M=*12, *SD=*8.06) and participants from different parental dyads (*M=*6.09, *SD=*6.55) differentiated between the number of years since occurrence of the loss. The mean age of the child at the time of death was 10.2 years (*SD*=9.4; range=4 days to 40 years).

The study was conducted according to specific ethical advice for clinical research with bereaved parents (Dyregrov, 2004; Hynson, Aroni, Bauld, & Sawyer, 2006). All participants provided written informed consent.

### Measures

*Meaning reconstruction* was assessed with a guided interview developed for this study, extending the approach of earlier research (e.g., Lehman, Wortman, & Williams, 1987; Lichtenthal et al., 2010; McIntosh, Silver, & Wortman, 1993) and building on the definitions from Davis et al. (1998). The resulting questions assessing sense-making are the following: (1) Have you been able to make sense of the loss?; (2) Have you questioned the meaning of life as a result of the loss?; and (3) Has your worldview changed since the loss? Benefit-finding was assessed with the following questions: (1) What does the death of your child mean for you and your life?; (2) Has your aim in life changed since the loss?; and (3) Do you think that it is possible to discover positive consequences even through loss? Please describe your personal experiences.

*Posttraumatic growth* was assessed with the Posttraumatic Growth Inventory (PTGI; Tedeschi & Calhoun, 1996), in German translation (Maercker & Langer, 2001). The PTGI measured positive changes among individuals who have experienced a highly stressful event. With 21 items, the PTGI measured five domain sub-scales of positive changes: Relating to Others, New Possibilities, Personal Strength, Spiritual Change, and Appreciation of Life on a scale ranging from 0 (*did not experience*) to 5 (*experienced to a very great degree*). Internal consistency was estimated at 0.90; test–retest reliability at a 2-month interval is 0.71 (Tedeschi & Calhoun, 1996).

*Complicated grief* was assessed with the shortened version (Maercker & Langner, 2002) of the Complicated Grief Module (CGM; Horowitz et al., 1997), which assesses symptoms of complicated grief noticed in the last month. It measures intrusion (*α*=0.88), avoidance (*α*=0.82), and failure to adapt (*α*=0.84) on 26 items, which could be evaluated on a four-point Likert-scale ranging from 0 (*not true*) to 3 (*total agreement*).

### Data analysis

#### Qualitative analysis

Oral material from conducted interviews has been transcribed using special software (f5 for mac) and subsequently content analyzed (without using specific software) (Mayring, 2010). The written material was organized into meaning units [segments of responses with either sense-making or benefit-finding as defined by Davis et al. (1998)]. We identified *N=*334 independent meaning units across participants’ responses. Analyzing and comparing these meaning units built the basis to: (1) assign meaning units to existing themes (Lichtenthal et al., 2010) when definition criteria (Tables 2 and 3, “coding definition”) were met; and (2) develop new themes to accurately picture the broad content of participants’ answers. It was determined that a new topic required more than one participant talking about it. Themes which were only mentioned by one participant and not already captured by Lichtenthal et al. (2010) were classified as “Other theme.” By doing it this way, one coder abstracted 39 content themes. The coder (first author) was double checked and supervised during the whole coding process by the second author. In order to enhance study reliability, Lichtenthal et al. (2010) were contacted to discuss ambiguities.

#### Quantitative analysis

All analyses were conducted with SPSS 19.0. Gender differences as well as differences between participants from the same and from different parental dyads were evaluated using *χ* ^2^-tests, *t*-tests, and Mann-Whitney-U-tests. Pearson's r-correlations were computed when all requirements, assessed with Lilliefors-corrected Kolmogorov-Smirnov-test and Levene-statistics, were met, and in all other cases, Spearman-correlations were used. Correlations were examined as one- or two-tailed tests depending on the hypotheses. All non-specified correlation analyses were two-tailed. To account for problems of non-independence, Mann-Whitney-U-tests, *t*-tests, and *χ* ^2^-tests were used to calculate possible differences between participants coming from the same and from different parental dyads.

After analyzing both data-sets independently, the results were merged by means of data transformation (Creswell & Zhang, 2009). Qualitative data was, therefore, numerically scaled, exhibiting the degree of sense-making and benefit-finding in numerical order (see Table 1), and establishing a continuous variable.

**Table 1:** Data transformation

Meaning reconstruction themes		Degree of sense-making/benefit-finding (numerical value)
No sense/no benefit	→	None (0)
No sense/no benefit combined with other sense-making or benefit-finding themes	→	Medium (1)
Sense-making or benefit-finding themes other than no sense/no benefit	→	Large (2)

## Results

We abstracted 20 sense-making themes, each characterizing ways in which the death was made comprehensible for the parents. The most common responses were thoughts encompassing the purpose of life and death, and were given by 50% of the participants. Answers described the early death in the context of the fulfillment of the child's purpose in life, such as, “He was very early mature, and so I think he was done.” Or the purpose was given as a result of the experience with the child. For example, a mother who lost her disabled daughter stated, “The purpose of why she was here became clear to me. Now, I have the experience to work with disabled children.” Other common responses were biological explanations of the death, stated by 43.3% of the participants, and beliefs about the existence of an afterlife, which were stated by 40% of the participants.

A total of 36.7% of the parents stated that they could not make any sense of the loss; however, most of these participants (86.2%) also mentioned other sense-making themes in the course of the interview. This finding was included in the development of two additional categories, one describing the difficulty of being able, and willing, to understand such a traumatic event despite having some explanation (“Little sense of meaning” 10%). The other category includes the strong will to believe in a sense of meaning, without being able to know or describe it in words (“Belief in a sense of meaning” 6.7%).

Another developed theme encompassed the understanding that one's identity as a parent could not be destroyed by death (“Identity,” 20%), for example, “The fact that I am a mother has not changed, my child is just somewhere else” (see Table 2).

**Table 2:** Sense-making themes and frequencies (participants often gave multiple answers)

Sense-making theme	Coding definition	% (n)
Purpose of life/death	Fulfillment of purpose of child's presence in the world and/or the consequences following for one's own life.	50.0 (15)
Biological explanation	Biological or medical explanations of the death.	43.3 (13)
Theme of an afterlife	Beliefs in the existence of an afterlife and/or the reunion with the dead child in the future.	40.0 (12)
No sense of meaning	Explicit response of “no” to sense-making question, or stating that such an event cannot be understood.	36.7 (11)
Human existence	Discussion about the imperfection of the world, the inevitability of death, or the fragility of life.	36.7 (11)
Fate	Death of the child attributed to fate, destiny, or a universal plan.	23.3 (7)
Child's behavior	Actions of the child believed to be related to the death.	20.0 (6)
*Identity*	*Understanding the death as an event that cannot destroy the identity of a mother or father as a parent of that child and/or the continuing bonds with the child*.	*20.0 (6)*
End of suffering	Discussion about the end of the child's physical or mental suffering as a result of the death.	13.3 (4)
*Probability*	*Death of the child discussed as a very unlikely event that happened to them nevertheless*.	*10.0 (3)*
*Foreshadowing*	*Descriptions of having foreseen the death of the child*.	*10.0 (3)*
*Challenge*	*Event seen as challenge in life and/or sense could be made in the way to deal with it*.	*10.0 (3)*
*Little sense of meaning*	*Uncertainty about being able to profoundly understand the event in a way that makes sense*.	*10.0 (3)*
*Gift*	*Viewing the time with the child as a gift and/or being deeply thankful for the time spent together*.	*10.0 (3)*
Parent's role	Belief that parent's own actions were related to the death.	6.7 (2)
*Belief in a sense of meaning*	*Strong belief that there is a deeper sense in this event that may or may not be revealed in the future*.	*6.7 (2)*
Information seeking	Discussion about obtaining information concerning the death in the context of understanding why the death occurred.	3.3 (1)
Random	Death seen as a random event.	3.3 (1)
Laws of physics	Laws of physics given as an explanation for the death.	3.3 (1)
God's will	Discussion of the death as an expression of God's will or plan.	–
Other themes	Other sense-making themes only one person mentioned.	10.0 (3)

*Note:* Italicized themes represent themes developed in the course of this study that were not already included in the themes of Lichtenthal et al. (2010).

In total, 19 benefit-finding themes emerged in parent's responses, of which the theme of personal improvement was most frequently named (46.7%). Responses concerning personal improvement were multifaceted and included, for example, describing personal growth, having a greater trust in life, being more tolerant, or developing personal potential. As one father stated, “I've learned to live, or rather, discovered a potential in me that I did not have before.” Other frequent themes were the description of changed priorities (43.3%) such as, “It is then no longer work, money, and material things that count, but concentrating on the essentials.”

A total of 36.7% of participants stated that they developed a heightened appreciation of life. A third of the participants (33.3%) described either a desire or concrete activities to help others in need, for example, “As a result of my experiences, I aim to build up a self-help group for parents and siblings.” Thirty percent of the participants also described feeling stronger, less afraid or better able to cope with difficult situations. For example, “I am now less afraid in life, especially concerning my own death.”

This feeling of acquiring a stronger ability to cope might also be a consequence of thinking that nothing worse could possibly happen. Such thoughts were included in the theme “Bitter benefit,” describing positive aspects that cannot be seen as a positive gain (13.3%). Another added theme (“Experience”), emphasized experiences with or following the loss (20% of the parents). For example, “In principle, it is a gift being able to have such an experience.” Some of the participants (10%) described a gain in freedom or time as a consequence of the loss.

A quarter of the participants (24.1%) stated explicitly “No” to the question of benefits or no further positive implications of their loss. At the same time, 57.1% of these parents also mentioned other themes determined as benefit-finding themes. This seeming ambivalence is illustrated by a mother who lost her 19-year-old son, “I think that the price is much too high. I don't want to learn, or mature. Maybe yes, one matures facing such an adversity. But it doesn't justify the death of someone” (see Table 3).

**Table 3:** Benefit-finding themes and frequencies (participants often gave multiple answers)

Benefit-finding theme	Coding definition	% (n)
Personal improvement	Themes of personal growth other than those detailed separately in this table (e.g., increased sensitivity), such as becoming wiser, more tolerant, etc.	46.7 (14)
Changed priorities	Changes in priorities or in the importance of how time is spent, or about what is distressing.	43.3 (13)
Appreciation of life	Heightened appreciation of life and of the moment, not taking anything for granted.	36.7 (11)
Helping others	Description of actions to help others or the desire to help others who have experienced losses or other painful events.	33.3 (10)
Stronger coping	Discussion of ways in which participants feel stronger, are better able to cope with difficult situations, or are generally less afraid, or are less afraid of specific issues, such as dying.	30.0 (9)
No benefit	Explicit response of “no” to benefit-finding question and/or stating that there is no positive implication of such a terrible loss.	26.7 (8)
Appreciation of others	Increased appreciation of others and/or heightened ability to express appreciation to others (also other children).	26.7 (8)
Increased sensitivity	Increase in perceptual sensitivity in general and/or empathy and compassion towards other human beings.	23.3 (7)
Valuable lessons	Lessons learned about life, death, relationships, etc.	23.3 (7)
*Experience*	*Having and being open for new experiences or discoveries*.	*20.0 (6)*
Education	Description of pursuing classes, or a (desired) change in careers.	16.7 (5)
Enhanced spirituality	Increases in spirituality or religiosity.	13.3 (4)
*Bitter benefits*	*Presumed positive, objective changes in life, which cannot really be seen as positive, such as there is nothing worse that could happen now*.	*13.3 (4)*
Increased empathy	Increased empathy for other bereaved parents and/or other suffering people.	10.0 (3)
Relationships developed	Strengthening of relationships and/or formation of new personal relationships.	10.0 (3)
*Freedom*	*Description of a gain in time or freedom in consequence of the loss*.	*10.0 (3)*
Lifestyle improvements	Positive changes in life such as ending a difficult relationship.	6.7 (2)
Support from others	Description of efforts made by family and/or friends following the loss.	3.3 (1)
Benefits to others	Discussion of how consequences of the child's death benefited others (e.g., organ donation) or the society.	–
Relationships to other children	Discussion of giving birth to or adopting other children, or an improvement in relationships with other children (see Appreciation of others).	_
Other themes	Other benefit-finding themes only one person mentioned.	10.0 (3)

*Note:* Italicized themes represent themes developed in the course of this study that were not already included in the themes of Lichtenthal et al. (2010).

Analysis revealed that more women were rating high on the CGM (*t=*−3.23; *p*<0.01). There were no gender differences in terms of posttraumatic growth (*t=*0.14; *p*=0.89), sense-making (*χ* ^2^=0.99; *p*=0.61), and benefit-finding (*χ* ^2^=0.08; *p*=0.96). A significant negative correlation between age and posttraumatic growth (*r=*−0.42; *p*<0.05), unaltered when controlled for the degree of benefit-finding (*r=*−0.33; *p*<0.05), but disappearing when controlled for sense-making (*r=*−0.19; *p*=0.32), was shown. A significant correlation was also found between age and complicated grief (*r*_(one-tailed)_=−0.33; *p*<0.05), unaltered when controlled for the degree of sense-making (*r=*−0.32; *p*=0.049) or benefit-finding (*r=*−0.34; *p*<0.05). There was no significant age difference between participants below and above the clinical cut-off (*t=*1.19; *p*=0.24).

Correlation analysis revealed a significant relationship between age and the degree of sense-making (*r*_*s*_=−0.53; *p*<0.01), whereas age and the degree of benefit-finding did not correlate significantly (*r*_*s*_=−0.27; *p*=0.15).

Participants from the same and from different parental dyads differentiated according to the number of years having elapsed since the loss, with a greater number of years having elapsed for participants from the same parental dyads (*M=*12, *SD*=8.06), compared to participants from different parental dyads (*M=*6.09, *SD*=6.55). Analysis revealed that the higher the number of years having elapsed since the loss, the lower the score in the CGM (*r*_(one-tailed)_=−0.49; *p*<0.01), and for participants above the clinical cut-off of complicated grief, less time had elapsed since the loss (*r*_pb_=−0.37; *p*=0.03). However, time had no significant relationship with posttraumatic growth (*r*_(one-tailed)_=−0.25; *p*=0.09). No relationship was found between either the degree of sense-making (*r*_s(one-tailed)_=−0.23; *p*=0.11) or the degree of benefit-finding (*r*_(one-tailed)_=−0.05; *p*=0.39) and time since the loss. Moreover, a negative correlation was found between posttraumatic growth and complicated grief for bereaved participants of fewer than 7 years (*r=*−0.52, *p*<0.05) (see Table 4).

**Table 4:** Measures of posttraumatic growth and complicated grief

	Total (*n=*29)	Men (*n=*9)	Women (*n=*20)	Gender differences *χ* ^2^/*t*/*u*-tests (*p*)
PGI, *M* (*SD*)	68.10 (16.99)	68.78 (17.87)	67.80 (17.05)	*t=*0.14 (.89)
CGM, *M* (*SD* **)**	28.72 (12.58)	19.00 (9.62)	33.10 (11.37)	*t=*−3.23 (.00)**
Sense-making				*χ* ^2^=0.99 (.61)
Benefit-finding				*χ* ^2^=0.08 (.96)

** *p*<0.01. PGI=Posttraumatic Growth Inventory; CGM=Complicated Grief Module.

Results revealed significant correlations between sense-making and posttraumatic growth (*r*_s_=0.54, *p*=0.00), as well as between benefit-finding and posttraumatic growth (*r*_s_=0.49, *p*=0.00). There was no statistically significant correlation between either sense-making (*r*_s_=0.08, *p*=0.34) or benefit-finding (*r*_*s*_=−0.07, *p*=0.35) with complicated grief. No significant correlation between posttraumatic growth and complicated grief was found (*r*=0.00, *p*=0.48) (see Table 5).

**Table 5:** Relations of meaning reconstruction, complicated grief and posttraumatic growth

	PGI	CGM	Sense-making	Benefit-finding
PGI	1	*r*=0.00 (0.48)	*r*_s_=0.54 (0.00)**	*r*_s_=0.49 (0.00)**
CGM	*r*=0.00 (.48)	1	*r*_s_=0.08 (0.34)	*r*_*s*_=−0.07 (0.35)
Sense-making	*r*_s_=0.54 (.00)**	*r*_*s*_=0.08 (0.34)	1	*r*_s_=0.18 (0.35)
Benefit-finding	*r*_s_=0.49 (.00)**	*r*_*s*_=−0.07 (0.35)	*r*_s_=0.18 (0.35)	1

** *p*<0.01. PGI=Posttraumatic Growth Inventory; CGM=Complicated Grief Module.

Analysis revealed that the correlation between sense-making and posttraumatic growth was especially high for objectively traumatic deaths (*r*_s_=0.67, *p*=0.00) and subjectively traumatic deaths (*r*_s_=0.63, *p*=0.00).

## Discussion

The primary goal of the present study was to scrutinize and expand meaning reconstruction themes identified by Lichtenthal et al. (2010) in a sample of bereaved parents with qualitative methods. Results revealed qualitative evidence of the comprehensive ways bereaved parents try to reconstruct meaning: 30 of 32 existing themes (Lichtenthal et al., 2010) were reproduced and 10 additional themes were identified.

Results showed that 82.2% of the participants noted at least one way they had made sense of their loss, whereas only 52.6% in the study of Lichtenthal et al. (2010) did so. This inconsistency may lie in the fact that meaning reconstruction requires time (Helgeson, Reynolds, & Tomich, 2006; Keesee et al., 2008; Murphy et al., 2003), which in contrast to this work, was not accounted for in the study of Lichtenthal et al. (2010).

The most common way of sense-making was the parent's preoccupation with the child's purpose in life, which often evoked the theme of their incontestable identity as a parent (Meert, Briller, Schim, Thurston, & Kabel, 2009). Current research increasingly recognizes this process of identification and retaining bonds with the deceased. For example, Ronen et al. (2009–2010) noted that identification with the deceased child differentiated between grieving bereaved parents and complicated grieving bereaved parents. However, under certain conditions continuing bonds to the deceased may also be maladaptive (Field, 2006), and being able to make sense of a loss might foster the adaptive nature of retaining bonds (Neimeyer, Baldwin, & Gillies, 2006) underlining the clinical importance of sense-making.

None of the 30 (German and Austrian) participants in the present study described understanding the child's death in the context of God's will, whereas this was the most frequently mentioned (17.9%) sense-making theme by predominantly US participants of Lichtenthal et al. study (2010). Other research in the Anglo-American area supports the importance of religious coping (e.g., Davis et al., 1998; McIntosh et al., 1993). The conflicting results might display a cultural difference between Europe and the US concerning the power of religious beliefs in explaining life and death (Walter, 2012): According to Pally (2005), religion plays an essential role in the US, whereas Western Europe is experiencing increasing criticism of institutional religion.

With respect to benefit-finding, comparisons showed that 73.7% of the participants in the study of Lichtenthal et al. (2010) offered at least one benefit in relation to their loss; that is, 89.7% of the participants in the present study did so. As for sense-making, these differences could be explained by the stricter time criteria imposed by this study.

Inconsistent with Lichtenthal et al. (2010) study, no one mentioned “benefits for other people” as consequence of the loss. Drawing on the theme definition (Lichtenthal et al., 2010), it is strongly related to the circumstances of the death, that is, to objective positive consequences such as organ donation. In this study, no such positive consequences for others arose, although some parents are engaged in processes to make that happen, which was included in the theme “helping others.” For example, one mother of a child dying as a result of substance abuse is engaging in the prevention of addiction.

The qualitative approach allowed revealing the struggle some parents experience with benefit-finding, such as the feeling of being forced into justifying their loss. A thought one mother describes as helping her to resolve this struggle speaks for itself:

No father or mother would ever say, “My child has died so that I could learn something.” Therefore, this idea of a heritage is also a proof that my child was here and things have changed. Children leave marks: If a child has been in a room, you notice—because they usually leave a mess (laugh). You know it, and it's the same with children who have died.

Generally, the qualitative data of the current work is an important contribution to existing research on meaning reconstruction, as the assessment was accomplished with elaborated in-depth interviews, assuring guidance and support in the discussion of such a sensitive topic (Dyregrov, 2004; Lichtenthal et al., 2010) as well as rich results. In contrast, most of the research to date assessed meaning reconstruction in writing and with fewer questions (e.g., Currier et al., 2006; Lichtenthal et al., 2010; Wheeler, 2001).

A secondary aim was to look for associations of meaning reconstruction with complicated grief and posttraumatic growth, with special attention focused on traumatic and unexpected losses. Referring to complicated grief the negative relationship to meaning reconstruction could not be supported either in general (Currier et al., 2006; Keesee et al., 2008; Lichtenthal et al., 2010, Neimeyer et al., 2006) or for traumatic loss (Murphy et al., 2003; Neimeyer et al., 2006). The reason for this discrepancy may lie in the smaller sample size of the current study, which is however comparable to other studies with bereaved parents involving oral interviews. (Barrera et al., 2009; Brabant, Forsyth, & McFarlain, 1997; Dyregrov & Dyregrov, 1999; Meert et al., 2009; Possick, Sadeh, & Shamai, 2008; Riches & Dawson, 1998).

Referring to posttraumatic growth, this study supports the notion that a positive relationship to meaning reconstruction exists (Calhoun et al., 2010, Gillies & Neimeyer, 2006; Triplett et al., 2012). Following the argument of Linley and Joseph (2011), we conclude that study inclusion criteria related to the time since the loss, ensured that the process of meaning reconstruction could already have led to find meaning associated with positive changes, in contrast to the searching phase. Along with Neimeyer, Prigerson and Davies (2002), it was shown that in the case of traumatic loss, sense-making was highly correlated with posttraumatic growth and at the same time seems to be more challenging for parents bereaved by traumatic loss (Lichtenthal, Neimeyer, Currier, Roberts, & Jordan, 2013).

### Limitations

Due to the small sample size, the self-selection of participants and the fact that most of the participants were female, the generalizability of the findings may be limited. Moreover, many participants came from a parental dyad, thus grieving for the common child, which could create a problem with non-independence (see “nested data” Wijngaards-de Meij et al., 2005).

## Conclusion

This work provides support for theoretical plurality regarding bereavement research and practice (Rosner, Kruse, & Hagl, 2010; Rosner, Pfoh, & Kotoučová, 2011) and underlines the value of modern models of grief (e.g., Gillies & Neimeyer, 2006; Janoff-Bulman, 1992; Klass, 1999).

Further, it underlines the importance of meaning-centered therapeutic techniques and the need for clinicians to address this process with an open mind, given the individuality of every loss experience; that is, to be prepared for spiritual thoughts as well as the need to function as interpreter between the client and a medical team, in order to enhance understanding.

Drawing on mentioned limitations, an essential next-step would be extensive qualitative research in order to develop a reliable instrument measuring meaning reconstruction. This would consequently allow for longitudinal studies in larger samples as well as the use of analysis allowing causal interpretations of findings.
